# Complete Genome Sequence of Klebsiella aerogenes Myophage Metamorpho

**DOI:** 10.1128/MRA.01420-20

**Published:** 2021-02-04

**Authors:** Kyra E. Groover, Kameron Garza, James Clark, Isla Hernandez, Jason J. Gill, Mei Liu

**Affiliations:** a Center for Phage Technology, Texas A&M University, College Station, Texas, USA; DOE Joint Genome Institute

## Abstract

The bacterium Klebsiella aerogenes is an opportunistic pathogen that often infects hospitalized patients and those who are immunocompromised. K. aerogenes in some cases can become resistant to antibiotic treatment. Being a potential therapeutic, Metamorpho is a T4-like myophage targeting K. aerogenes.

## ANNOUNCEMENT

Klebsiella aerogenes is an opportunistic pathogen that is generally associated with urinary tract infections, abdominal infections, septicemia, and pneumonia, with a majority of infections associated with this pathogen being hospital acquired (1). There is a high incidence of resistance to β-lactam antibiotics in K. aerogenes (1). Phage Metamorpho is able to lyse and kill K. aerogenes, and its annotated genome is described here.

Metamorpho was isolated from wastewater collected from the wastewater plant in Madisonville, Texas, using K. aerogenes strain ATCC 13048 with the soft-agar overlay method (2). The phage was propagated at 37°C on LB agar seeded with the host bacterial strain. The phage morphology was visualized via transmission electron microscopy (TEM) of virions stained with 2% (wt/vol) uranyl acetate (3) at the Texas A&M Microscopy and Imaging Center. Phage DNA was extracted using the Promega Wizard DNA extraction system following the steps described previously (4) and prepared for sequencing using the Illumina Nextera kit with 300-bp inserts. The prepared DNA was subjected to sequencing with a MiSeq system using 300-cycle v2 chemistry. The sequences returned were checked for quality by FastQC (www.bioinformatics.babraham.ac.uk/projects/fastqc). The library index containing this phage generated 686,406 sequencing reads, and ∼5% of the total reads were assembled into SPAdes v3.5.0 (3) into a contig corresponding to this phage with 10× coverage. The genome was closed using PCR, with the primers 5′-GCTATTCTATCCCAACGGTCAG-3′ and 5′-GAATAGGATCAACCGAGTTACCG-3′, and Sanger sequencing of the PCR product. Structural annotation was conducted with GLIMMER v3 (5) and MetaGeneAnnotator v1.0 (6) for identification of putative genes. ARAGORN v2.36 (7) was used to identify tRNA genes. Protein functions were predicted by InterProScan v5.33 (8), BLAST v2.9.0 (9) with the NCBI nonredundant and Swiss-Prot databases (10), TMHMM v2.0 (11), LipoP (12), and HHpred (13) with default settings. The whole-genome sequence was compared to those of other phages via progressiveMauve v2.4 (14). All of the tools used for analysis were run on the Center for Phage Technology (CPT) Galaxy and Apollo interfaces (15–17) with default settings (https://cpt.tamu.edu/galaxy-pub).

The complete genome length of phage Metamorpho is 171,475 bp. It has a lower GC content (38.5%) than its host (54.1%) (18). A total of 287 protein-coding genes and 15 tRNAs were predicted, for a coding density of 94.0%. Metamorpho is most closely related to other T4-like *Klebsiella* phages; the most notable are JD18 (GenBank accession number KT239446), Mineola (MH333064), and KP1 (MG751100). Metamorpho is also related to the canonical Escherichia coli phage T4, with 75.7% DNA sequence similarity to T4 by BLASTn analysis and 177 proteins shared with T4 by BLASTp (E <10^−5^). Not surprisingly, Metamorpho showed T4-like myophage TEM morphology (image not shown). Metamorpho was predicted by PhageTerm (19) to package its DNA by headful packaging, and the genome was reopened at the *rII* locus to retain synteny with other T4-like phages. Metamorpho gp163 is a homolog of T4 protein e.6, which is conserved in a number of T4-like phages. Analysis of gp163 with HHpred indicated a high-quality alignment (99.9%) with E. coli phage shock protein PspA (4WHE_A), which is associated with the cellular stress response (20).

### Data availability.

The genome sequence of phage Metamorpho was deposited under GenBank accession number MT701588 and BioSample accession number SAMN14609635. The BioProject accession number is PRJNA222858, and the SRA accession number is SRR11558337.
